# C3: connect separate connected components to form a succinct disease module

**DOI:** 10.1186/s12859-020-03769-y

**Published:** 2020-10-02

**Authors:** Bingbo Wang, Jie Hu, Yajun Wang, Chenxing Zhang, Yuanjun Zhou, Liang Yu, Xingli Guo, Lin Gao, Yunru Chen

**Affiliations:** 1 School of Computer Science and Technology, Xidian University, Xi’an, People’s Republic of China; 2 School of Humanities and Foreign Languages, Xi’an University of Technology, Xi’an, People’s Republic of China; 3 The First Affiliated Hospital of Xi’an Jiaotong University, Xi’an, People’s Republic of China

**Keywords:** Disease module, Connectivity pattern, Biological network

## Abstract

**Background:**

Precise disease module is conducive to understanding the molecular mechanism of disease causation and identifying drug targets. However, due to the fragmentization of disease module in incomplete human interactome, how to determine connectivity pattern and detect a complete neighbourhood of disease based on this is still an open question.

**Results:**

In this paper, we perform exploratory analysis leading to an important observation that through a few intermediate nodes, most separate connected components formed by disease-associated proteins can be effectively connected and eventually form a complete disease module. And based on the topological properties of these intermediate nodes, we propose a connect separate connected components (C3) method to detect a succinct disease module by introducing a relatively small number of intermediate nodes, which allows us to obtain more pure disease module than other methods. Then we apply C3 across a large corpus of diseases to validate this connectivity pattern of disease module. Furthermore, the connectivity of the perturbed genes in multi-omics data such as The Cancer Genome Atlas also fits this pattern.

**Conclusions:**

C3 tool is not only useful in detecting a clearly-defined connected disease neighbourhood of 299 diseases and cancer with multi-omics data, but also helpful in better understanding the interconnection of phenotypically related genes in different omics data and studying complex pathological processes.

## Background

Most complex human diseases are rarely the consequence of the abnormal activity of a single gene product. Conversely, they involve the activities of multiple interacting genes, yet many of which are not defective [1]. Hence, it is crucial to study the relationship between genotypes and phenotypes to unlock the genetic origins of complex diseases [2–6] in the context of human interactome (molecular interactions networks, nodes are proteins and edges are observation and inference from their interactions). Driven by high-throughput interactome mapping efforts [7] and the genome-wide genetic association data [8], previous work has begun to unveil the relationships between different phenotypes based on network. The most important concept that emerges from this is the “disease module”. Disease-associated proteins, (DAPs, encoded by disease genes) for a specific disease phenotype show a tendency to cluster in the same network neighbourhood [3]. Topological properties of disease module in the interactome underlie all network-based prioritization tools for studying human diseases. Topological characteristics of existing studies include: specific phenotype-related DAPs frequently form a well-connected component rather than locally dense communities [9]; most disease genes show no tendency to encode hub proteins, but rather are located in the functional periphery of interactome [1]; furthermore, the position of each disease module relative to other modules in the interactome implies its phenotypic similarity to other diseases [10]; and recently, Agrawal et al. [11] have found that DAPs of a single disease tend to form many separate connected components in the network. Their research also shows that higher-order network structures such as small subgraphs of the pathway can provide a promising direction for the development of new methods.

Disease module helps to uncover the molecular mechanism of disease causation, identify new disease genes and pathways and drug targets [9, 12–18]. For example, Sharma et al. [18] have identified the disease module of asthma and verified its functional and pathophysiological correlation using both computational and experimental approaches. They found that the asthma disease module had a modest GWAS *p* values against the background of random variation, and there are differentially expressed genes in normal and asthmatic fibroblast cells treated with an asthma-specific drug. The asthma module also contains immune response mechanisms that are shared by other immune-related disease modules.

Numerous computational methods have been developed for the accurate identification of disease module to fully explain the molecular mechanism of most human diseases. With regard to the fragmentation of disease module [10], more than 80% of DAPs on average are shown to be scattered, forming many separate connected components (SCCs), this pattern is applicable to more than 90% diseases [19]. A disease module does not correspond to a single well-connected component as an observable module in present incomplete interactome. Therefore, many methods [9, 14, 20, 21] used for searching for DAPs-intensive clusters or communities within the interactome might have limitations in discovering disease modules. Such as DIAMOnD [9], the most advanced algorithm, is used to analyze the connectivity patterns of DAPs and form a disease module with a large number of intermediate proteins, which are known as DIAMOnD proteins. Although DIAMOnD can generate internal interconnected module for a specific disease, unfavourably, on average it introduces 200 DIAMOnD proteins, but only connects about average 50% DAPs of 299 diseases from Online Mendelian Inheritance in Man (OMIM [22]) and Genome-Wide Association Studies (GWAS [8]) databases. Many of the introduced DIAMOnD proteins are unnecessary for precisely detecting disease modules, resulting in enormous disease module sizes (average size of 400 proteins, i.e. the disease module has been magnified 4 times), ultimately the analysis of 299 disease relationships based on such modules is ambiguous. Another algorithm is based on the Steiner tree, the retrieval of a minimum size sub-graph leads to the classic Steiner tree problem, which is known as NP-complete [23], such as a heuristic method based on the minimum spanning tree approximation, the time complexity of the entire algorithm is about O(n^3^). This is impractical to detect disease modules in a large human interactome of more than 13,000 proteins, as it is a time-consuming process of finding the optimal connected components. On the other hand, any remote nodes can be connected through a Steiner tree, the connection properties of the disease module are therefore hindered by these problems, which may lead to the misunderstanding of the topological characteristics of DAPs and disease modules. Overall, despite many methods, the analysis of connectivity pattern of DAPs are still an unsolved problem.

In this paper we proposed a topological-based method to connect separate connected components (CCC, C3) in order to form a succinct disease module. We showed that SCCs formed by DAPs can be effectively connected through a few intermediate proteins and interactions in the network, which can statistically significantly reduce the number of fragments of a disease module. The specific process is as follows. First, we quantified the roles of proteins and interactions in connecting SCCs, i.e. by importing the intermediate proteins and interactions, C3 detects observable disease modules for DAPs of 299 disease phenotypes from the OMIM [22] and GWAS [8] databases. Next, we described the characteristics of the detected disease modules, we verified the succinctness and connectivity significance of C3 module by comparing this method with DIAMOnD and random simulations results. C3 module is a well-connected succinct disease module with small-size and dominated by DAPs. Additionally, we explored their relative contributions to disease pathogenesis in some case studies. And we presented evidence suggesting that even for DAPs from multi-omics data in The Cancer Genome Atlas (TCGA [24]), C3 worked as a practical strategy for understanding interrelationships between mRNA expression, mutation, DNA methylation and copy number variation, which further validated the biological significance of the disease module we obtained.

## Results

### Connectivity pattern of disease module

**Fig. 1:**
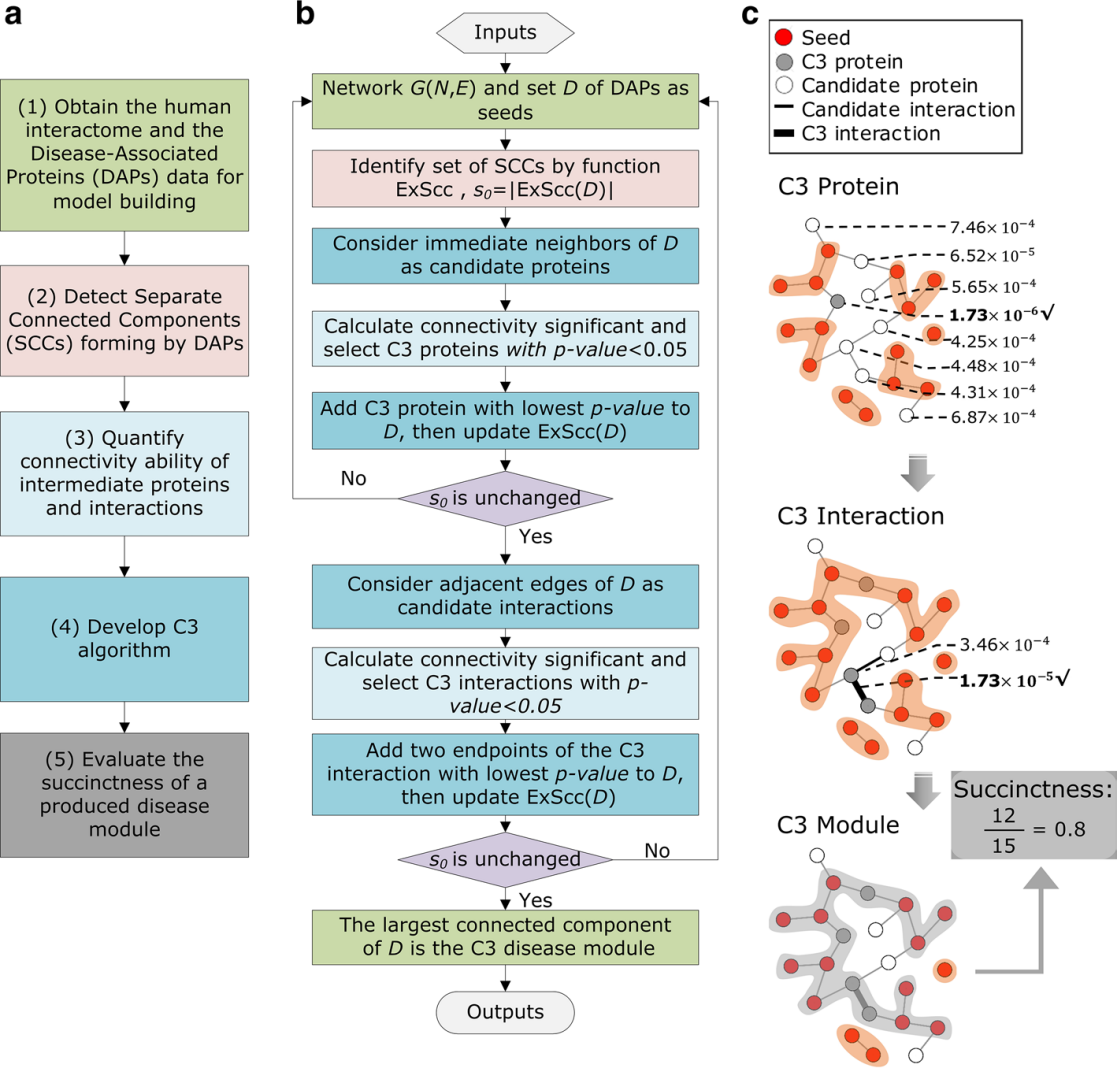
Connectivity pattern of disease module. **a** The flow framework of C3 Method. (i) Obtain the human interactome and the DAPs data for model building; (ii) Detect SCCs forming by DAPs; (iii) Quantify connectivity ability of intermediate proteins and interactions; (iv) Develop C3 algorithm; (v) Evaluate the succinctness of a produced disease module. **b** The detailed flow chart of the C3 algorithm. (i) The SCCs are identified for all seeds (set *D*) in network $G(N,E)$, forming a connected components set of SCCs with size ${s}_{0}$. (ii) The connectivity significant *p* values of candidate proteins (immediate neighbors proteins of *D*) are calculated and ranked, *p* value < 0.05 and the lowest protein is added to *D*, then update the set of SCCs with size ${s}_{0}$. (iii) The connectivity significant *p* values of candidate interactions (adjacent edges of *D*) are calculated and ranked, *p* value < 0.05 and two endpoints proteins of the lowest interaction are added to *D*, then update the set of SCCs with size ${s}_{0}$. (iv) Steps (i)–(iii) are repeated until there is no protein or interaction with *p* value < 0.05 can connect at least two SCCs, i.e. ${s}_{0}$ is unchanged, the largest connected component of *D* is the C3 disease module. **c** schematic diagram of C3 protein (gray dot) and C3 interaction (thick black line), after adding C3 proteins and C3 interactions, we get the C3 disease module (gray shadow). The yellow shadows are SCCs. Red and white dots represent seeds and candidate proteins respectively, and thin black lines represent candidate interactions. In addition, the fraction of the connected seeds in C3 module divided by the total number of seeds is defined as succinctness

To explore the connectivity pattern of disease module, we described our framework (Fig. 1a) as follows: (1) the human interactome and the Disease-Associated Proteins (DAPs) data for model building, (2) detection of Separate Connected Components (SCCs) forming by DAPs in the interactome, (3) quantification index of the connectivity ability of intermediate proteins, (4) greedy process of C3 algorithm, and (5) quantification of succinctness of final disease module.

We detected the connectivity pattern of disease module in a comprehensive list of experimental records of molecular interactions in human cells compiled by Menche et al. [10] (see Methods). In total, we obtained 141,296 interactions among 13,460 proteins. This human interactome can be modeled as an undirected network $G(N,E)$, where *N* is the proteins set and *E* is the interactions set. The known DAPs of 299 diseases are gathered from OMIM [22] and GWAS [8] databases (see Methods), of which 2436 are in *N* and associated with one or more diseases. We call these 2436 DAPs “*seeds*” in our subsequent algorithm process.

The SCCs forming by DAPs in *G* are identified by a tool networkx [25] in python which can export all the connected components of a given node set. We used a function ExScc (see Methods) to export the set of SCCs of a given seed set in our method. Based on this, the connectivity patterns of 299 typical complex diseases in a curated list are studied (see results in Additional file 1).

To validate whether there are some intermediate proteins or interactions can statistically significantly reduce the number of fragments of a disease module or not, we used hypergeometric *p* value to quantify their ability in connecting SCCs of DAPs. The *p* value evaluates whether a certain intermediate node or edge can connect more SCCs than expected (see Methods). We found that there are certainly some intermediates can significantly connect SCCs and reduced the number of them (*p* value < 0.05, connectivity is significant). A “*C3 protein*” or a “*C3 interaction”* is defined as a node or an edge with *p* value < 0.05 and lowest (Fig. 1b, c). The C3 proteins and the pair of proteins occupying the endpoint of C3 interactions are collectively referred to as “*C3 proteins*” in our functional analysis below. Then we formulated the disease module identification problem as a C3 proteins detection problem: *Succinct disease module detection problem*. Given a network $G(N,E)$ and a seed set *D*, ExScc(*D*) is a function which can export the set of SCCs of *D*, find a node set *C* with minimum *size*, such that $\forall c\in C$, *c* is a C3 protein, and $\underset{C}{\hbox{min}}\left|\hbox{ExScc}(D\cup C)\right|$. The initial seed set *D* forms lots of SCCs, and presents a large $\left|\hbox{ExScc}(D)\right|$, the size of SCCs will significantly decrease as introducing C3 protein set *C* into disease module. A small $\left|\hbox{ExScc}(D\cup C)\right|$ means that there are C3 proteins can significantly reduce the number of fragments of a disease module. Extremely $\left|\hbox{ExScc}(D\cup C)\right|=1$ indicates that all the DAPs can be connected by C3 proteins into one component in the network. Then we developed a greedy process (C3 algorithm in Fig. 1b, see Methods for details) to efficiently solve this problem, by importing C3 proteins and C3 interactions iteratively, and eventually a well-connected disease module becomes observable (Fig. 1c). Specifically the largest connected component (LCC) of $D\cup C$ are named *C3 module*.

Furthermore, previous methods such as DIAMOnD [9], also construct a disease module by introducing some intermediate proteins. Disease Module is the LCC of a union set of DAPs and intermediate proteins. It is obvious that more DAPs are connected by intermediate proteins, the final disease module are more dominated by known DAPs. Therefore, we proposed the fraction of connnected DAPs in the disease module ($|{\text{D}} \cap {\text{Dm}}|/|{\text{D}}|$) as an index to quantify the succinctness of a produced disease module *Dm* (Fig. 1c). The succinctness can be used to compare the purity between results of different methods. It is a value between 0 and 1, and the closer to 1, the more succinct the disease module is.

### Succinctness and connectivity significance of the C3 module

According to disease module hypothesis, genes associated with a specific disease phenotype tend to cluster in the same network neighborhood, usually referred to as a “*disease module*”. Furthermore, the location of each disease module relative to others in the network implies its phenotypic similarity to other diseases [10], and the position of the disease module in the network is of great importance. Through a basic easy-to-understand detection process, we could obtain C3 disease module, C3 proteins and C3 interactions (Fig. 1c) for a particular disease (see Methods). In DIAMOnD disease module, due to the large number of the DIAMOnD proteins, DIAMOnD module is a huge one dominated by DIAMOnD proteins, it would bring many false signals to the disease module. Comparatively, C3 disease module imports a small amount of C3 proteins and is dominated by DAPs, the C3 disease module can accurately pinpoint the area affected by the disease on the network and provide us with more accurate disease signals. Therefore, a succinct disease module is essential for studying disease occurrence and treatment.

**Fig. 2:**
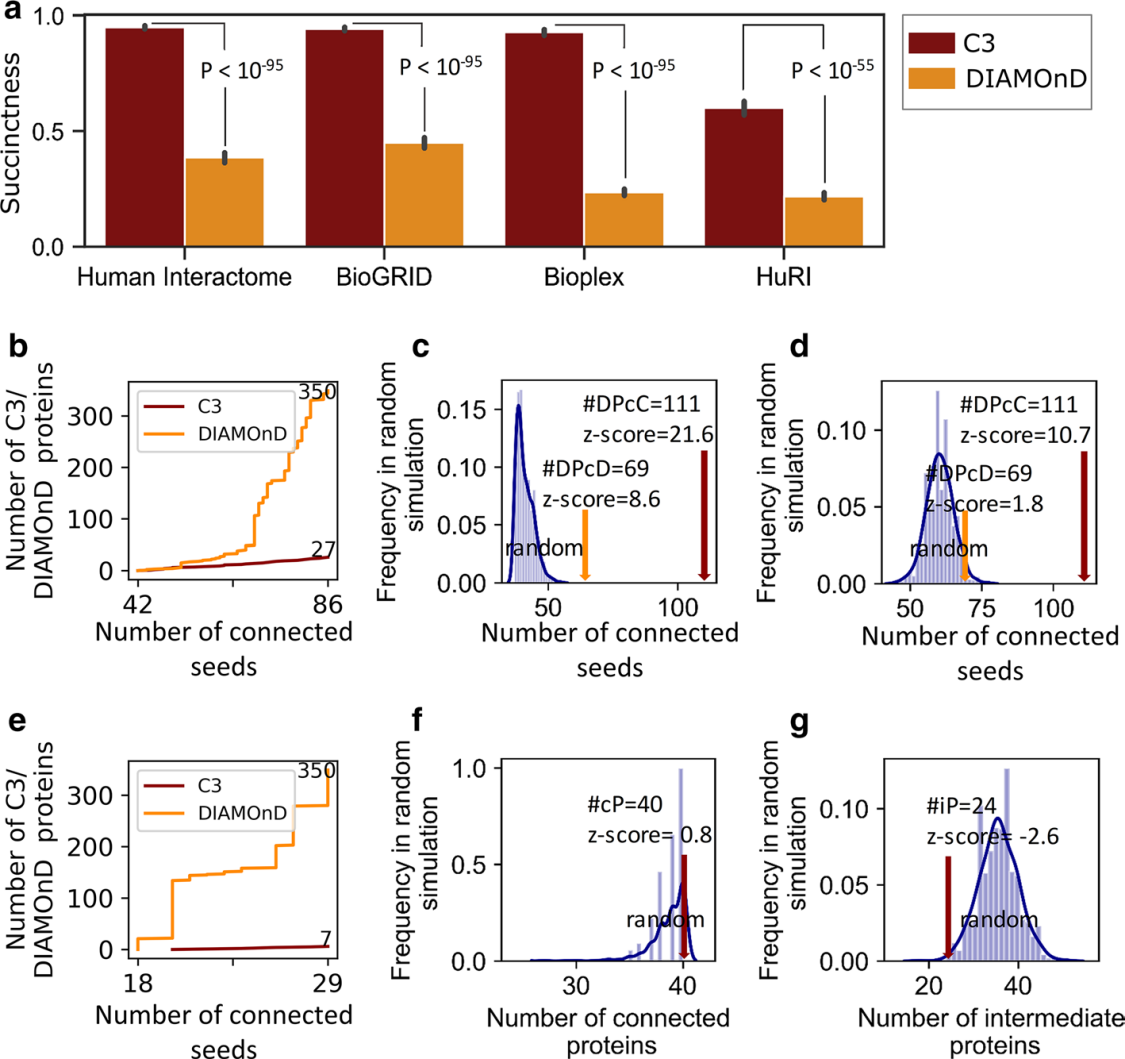
Succinctness and connectivity significance of the C3 module. **a** The succinctness of the C3 and DIAMOnD modules of 299 diseases in different networks, and *p* value is obtained by wilcoxon rank sum test. **b**–**d** For asthma, **b** when the modules obtained by DIAMOnD and C3 contain 86 seeds at the same time, red line indicates that only 27 C3 proteins are used in the C3 module, while yellow line indicates that 350 DIAMOnD proteins are used in the DIAMOnD module. **c** Comparison of the number of Disease Proteins contained in DIAMOnD module (DPcD) and C3 module (DPcC) with 1000 random simulations. Completely randomly-constructed modules contain an average of 44.9 ± 3.2 seeds, which is significantly lower than the value in the real disease module. And DIAMOnD module containing 69 seeds (z-score = 8.6) is lower than C3 module with 111 seeds (z-score = 21.6). **d** Similar to that in (**c**), but intermediate proteins are not chosen completely at random, but only from the immediate neighbor of seeds. The random modules contain 60.3 ± 4.7 seeds, which is again significantly lower than in the real disease module. And DIAMOnD module containing 69 seeds (z-score = 1.8) is also lower than C3 module with 111 seeds (z-score = 10.7). **e**–**g** With BRCA there are 40 DAPs (seeds), **e** Same as B, when DIAMOnD and C3 module contain 29 seeds at the same time, only 7 C3 proteins are used in the C3 module while 350 DIAMOnD proteins are used in the DIAMOnD module. Comparison of the number of connected Proteins (cP) (**f**) and the number of intermediate Proteins (iP) (**g**) in the final C3 module with the number obtained from 1000 random simulations. Randomly 40 proteins are selected with the same seed degrees each time. The average number of connected seeds is 38.7 ± 1.6, while C3 connected all 40 BRCA proteins. The number of intermediate proteins used on average is 35.6 ± 4.4, and only 24 C3 proteins are used in BRCA C3 module

As mentioned above, the 299 disease modules obtained by the DIAMOnD algorithm are redundant. We compared the C3 to the DIAMOnD algorithm on a data platform that is more conducive to DIAMOnD using the DIAMOnD supporting data, which includes: human interactome network and the curated list of 299 diseases (see Methods). We found that in human interactome C3 can effectively connect more seeds with a *p* value of 3.94e−99 (wilcoxon rank sum test) and its succinctness is about 95% while the succinctness of DIAMOnD is about 39% by using the same intermediate proteins. In addition, we used different types of systematic networks BioGRID, Bioplex and HuRI. Network information is shown in Table 1. In different types of networks, when the same intermediate proteins are imported, C3 can more effectively connect more seeds than DIAMOnD, and the differences in *p* values of succinctness are respectively 5.80e−98, 4.09e−96, 1.58e−57 (Fig. 2a). This fully demonstrates the succinctness of the C3 module.

**Table 1:** Network information

Network	Source	#Nodes #Edges	Mean degree	Diameter	Clustering coefficient
Human Interactome	Menche et al. [10]	13,460 141,296	20.6	13	0.17
BioGRID	https://thebiogrid.org/	17,741 338,793	37.9	8	0.12
Bioplex	https://bioplex.hms.harvard.edu/	10,961 56,553	10.3	11	0.09
HuRI	https://interactome.dfci.harvard.edu/H_sapiens/	7,352 38,414	10.3	11	0.06

Then we chose asthma and BReast CAncer (BRCA) as examples for detailed analysis (Fig. 2b–g). For asthma, the same 86 asthma seeds are connected by the DIAMOnD and C3, C3 proteins require 27 intermediate proteins while DIAMOnD uses 350 intermediate proteins, which is about 13 times that of C3. The same is for BRCA. To connect 29 BRCA seeds, 7 C3 proteins and 350 DIAMOnD proteins are required. The number of the imported DIAMOnD proteins is about 50 times that of C3 (Fig. 2b, e). Therefore, using fewer C3 proteins can connect the same number of the seeds, further proving the advantages of C3 method in connecting seeds. C3 method is equally applicable to other diseases (Fig. 2a).

Connectivity significance refers to the ubiquitousness of C3 proteins and C3 interactions in the network. They can significantly connect SCCs of seeds, i.e., they can play a role in the connection of disease modules. To test whether the observed disease modules represent non-random aggregation of DAPs, we performed randomized experiments on asthma and BRCA (see Methods). For asthma, we found that completely random modules of the same size (add 100 intermediate proteins as DIAMOnD) contained an average of 44.9 ± 3.2 seeds, which was significantly lower than the number of seeds in the real disease module, both DIAMOnD module (contains 69 seeds, z-score = 8.6) and C3 module (contains 111 seeds with only 61 C3 proteins, z-score = 21.6) (Fig. 2c), even if the proteins are randomly chosen only from the immediate neighbors of seeds, it is observed that the resulting modules contain only 60.3 ± 4.7 seeds, which is again significantly lower compared with the DIAMOnD module (z-score = 1.8) and C3 module (z-score = 10.7) (Fig. 2d). For the 40 DAPs of BRCA, we randomly selected 40 proteins with the same seed degree for random experiments. We used 35.6 ± 4.4 intermediate proteins to connect 38.7 ± 1.6 seeds which were randomly selected. It is shown that C3 not only connects all 40 seeds (z-score = 0.8) and uses fewer intermediate proteins (z-score = − 2.6) compared to random simulations (Fig. 2f, g). These differences successfully demonstrate the connectivity significance of the disease module in the interactome, indicating the significant connecting tendency of DAPs. And the C3 disease module is superior to DIAMOnD in revealing the connection patterns of DAPs in the interactome.

### C3 module in asthma

**Fig. 3:**
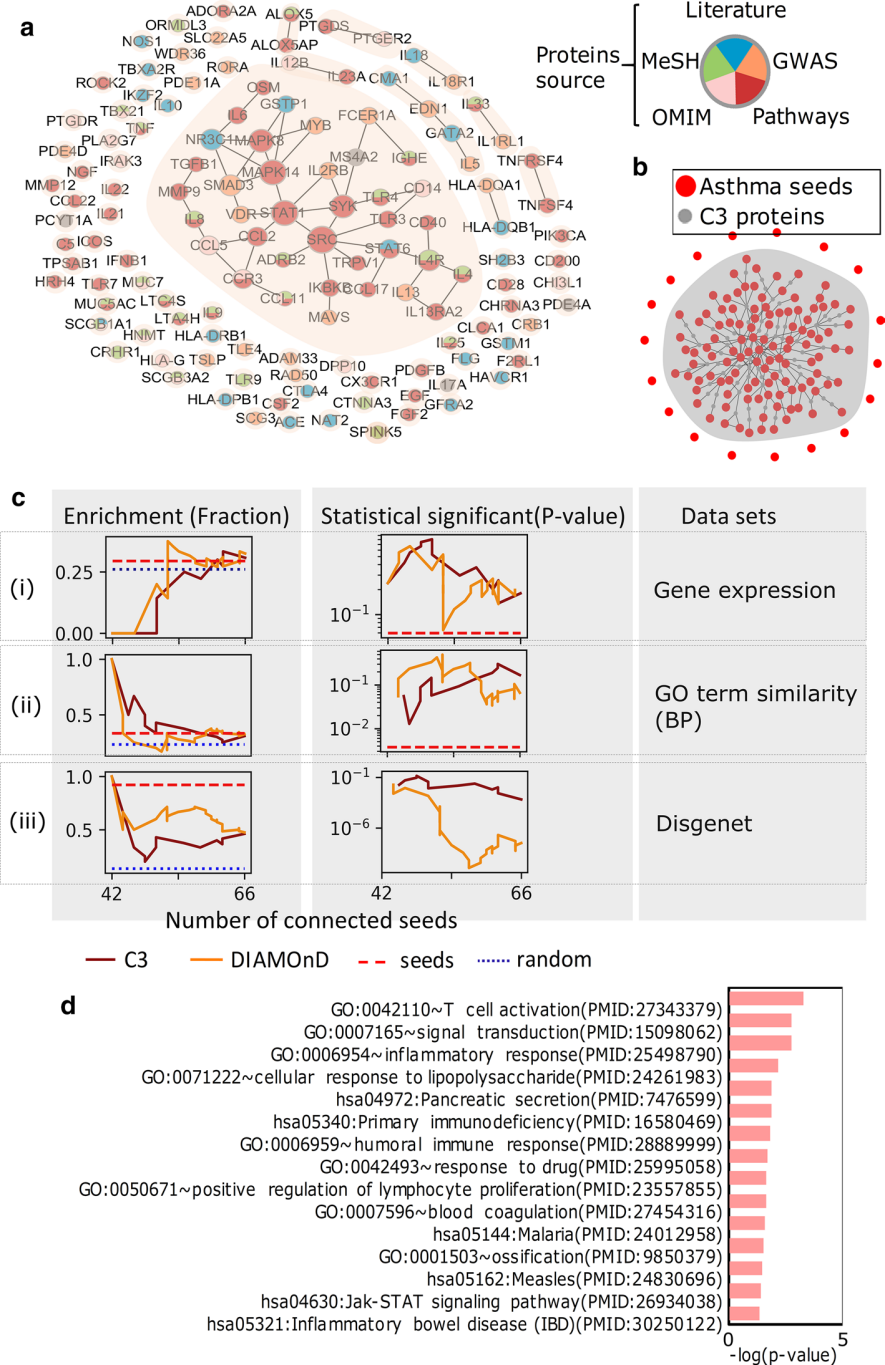
The visualization and biological explanation of C3 module for asthma. **a** Subgraph of the full interactome showing the connections among the asthma DAPs. Asthma DAPs are scattered within 83 SCCs in the interactome (shown in shades). The colors of the nodes indicate the source of each seed. There are five sources: (i) Literature; (ii) GWAS; (iii) OMIM; (iv) MeSH; (v) Pathways. **b** C3 module of Asthma. There are 111 seeds (red nodes) and 61 C3 proteins (grey nodes) in the module. The interaction between them is also indicated in the module. **c** Biological interpretation of C3 module for asthma. We consider three verification datasets: (i) Gene expression of asthma-specific compiled from eight sources; (ii) GO term similarity (BP); (iii) Disgenet. Enrichment shows the fraction of C3 (Deep red line), DIAMOnD (green line), seeds (short dotted red line) and random (Dotted blue line) proteins in different validation datasets, statistical significance shows the corresponding statistical test *p* values, which are derived from the hypergeometric. (D) Functional annotation of GO term (BP) and KEGG pathway (*p* value < 0.01) of C3 proteins using DAVID. The pink bar indicates the − log_10_ operation on the enriched *p* value

We have obtained C3 modules of 299 diseases. Can these modules effectively characterize the corresponding diseases? In the following, we analyzed asthma C3 module and compared it with DIAMOnD module. The asthma test mentioned above is based on 139 DAPs from five sources: (1) Literature; (2) GWAS; (3) OMIM; (4) MeSH; (5) Pathways [18], 129 (92.8%) of which are in the human interactome network. Then we projected them onto the interactome and obtained the SCCs (Fig. 3a). It was observed that the seeds formed a large number of SCCs (83 SCCs) in the real interactome. Then, C3 method was used to connect these SCCs to obtain a well-connected module of asthma. We used 61 C3 proteins to well connect 65 (78%) SCCs, inferring that the SCCs tend to be connected rather than dispersed (Fig. 3b). In addition, we need biological enrichment analysis to verify the significance of this asthma C3 module. For unconfirmed C3 proteins in this module, its relationship with asthma has not yet been found, and we have suggested this possibility through enrichment analysis and provided clues for the diagnosis and treatment of asthma. We used three different asthma-specific validation data: (1) Gene expression of asthma-specific compiled from eight sources; (2) Gene Ontology (GO) term similarity (biological processes, BP); (3) Disgenet (see Methods and Fig. 3c). The similarity of GO term is calculated by using the tool GOSemSim [26]. In these three assessments, we observed that enrichment of C3 and DIAMOnD proteins can reach the same level. And the level of enrichment *p* value of C3 is comparable to DIAMOnD and even better sometimes. These three enrichment analyses show that C3 proteins have biological significance, and it is speculated that it is biologically related to asthma, so the 61 C3 proteins and the connected seeds form C3 module, which is a reliable asthma module.

Furthermore, in order to better dig into the role of C3 proteins in the underlying disease pathways, we used DAVID [27] (https://david.ncifcrf.gov/) to study the functional annotation of the 61 C3 proteins in asthma (Fig. 3d). Here we focused on the function annotations of GO terms (BP) and KEGG pathway. We found that C3 proteins enrichment in some pathways has a lower *p* value, which has been reported to be associated with asthma (PMID), such as: neuropeptide Y may promote TH2 inflammatory response in asthma [28], signal transduction of IL-13 plays a role in the pathogenesis of bronchial asthma [29], humoral immune responses during asthma [30]. And in Table 2, we present in detail the enrichment function and enriched C3 genes as well as the PMID verified by the literature. Some C3 proteins are highly associated with asthma, such as: CD80 influences synthesis of IL-4 and IFN in non-specific bronchial asthma [31], mutations in IL12A influence cockroach allergy among children with asthma [32], ADA polymorphisms are related to asthma [33]. It indicates that the C3 module has biological significance. To be specific, it can explain the disease mechanism and help predict potential pathogenic genes or novel drug targets.

**Table 2:** GO term and KEGG pathway functional annotation of C3 proteins and literature-validated PMID of C3 genes associated with asthma

GO term/KEGG	#Genes	Gene(PMID)
GO:0042110	4	CD80(PMID:14692664), ADA(PMID:16754522), FOXP3(PMID:26646898)
GO:0007165	12	CRH(PMID:10423904), ERBB3(PMID:11799369), IL9R(PMID:11039580), MYD88(PMID:27289030), PTGES(PMID:27611488)
GO:0006954	7	CCR4(PMID:29118379), CRH(PMID:10423904), IL37(PMID:29137646), MYD88(PMID:27289030)
GO:0071222	4	CD80(PMID:14692664), B2M(PMID:26051416), IL12A(PMID:18671862), TFPI(PMID:16095153)
hsa05340	3	ADA(PMID:16754522)
GO:0006959	3	EBI3(PMID:28553015), LTF(PMID:22837640)
GO:0042493	5	ADA(PMID:16754522), B2M(PMID:26051416), CRH(PMID:10423904)
GO:0050671	2	IL12A(PMID:18671862), MYD88(PMID:27289030)
GO:0007596	4	PRTN3(PMID:29665127), TFPI(PMID:16095153)
hsa05144	3	IL12A(PMID:18671862), MYD88(PMID:27289030)
GO:0001503	3	SP3(PMID:23057572), LTF(PMID:22837640)
hsa05162	4	IL12A(PMID:18671862), MYD88(PMID:27289030)
hsa04630	4	IL12A(PMID:18671862), IL9R(PMID:11039580)

### C3 module in TCGA multi-omics data of BRCA

**Fig. 4:**
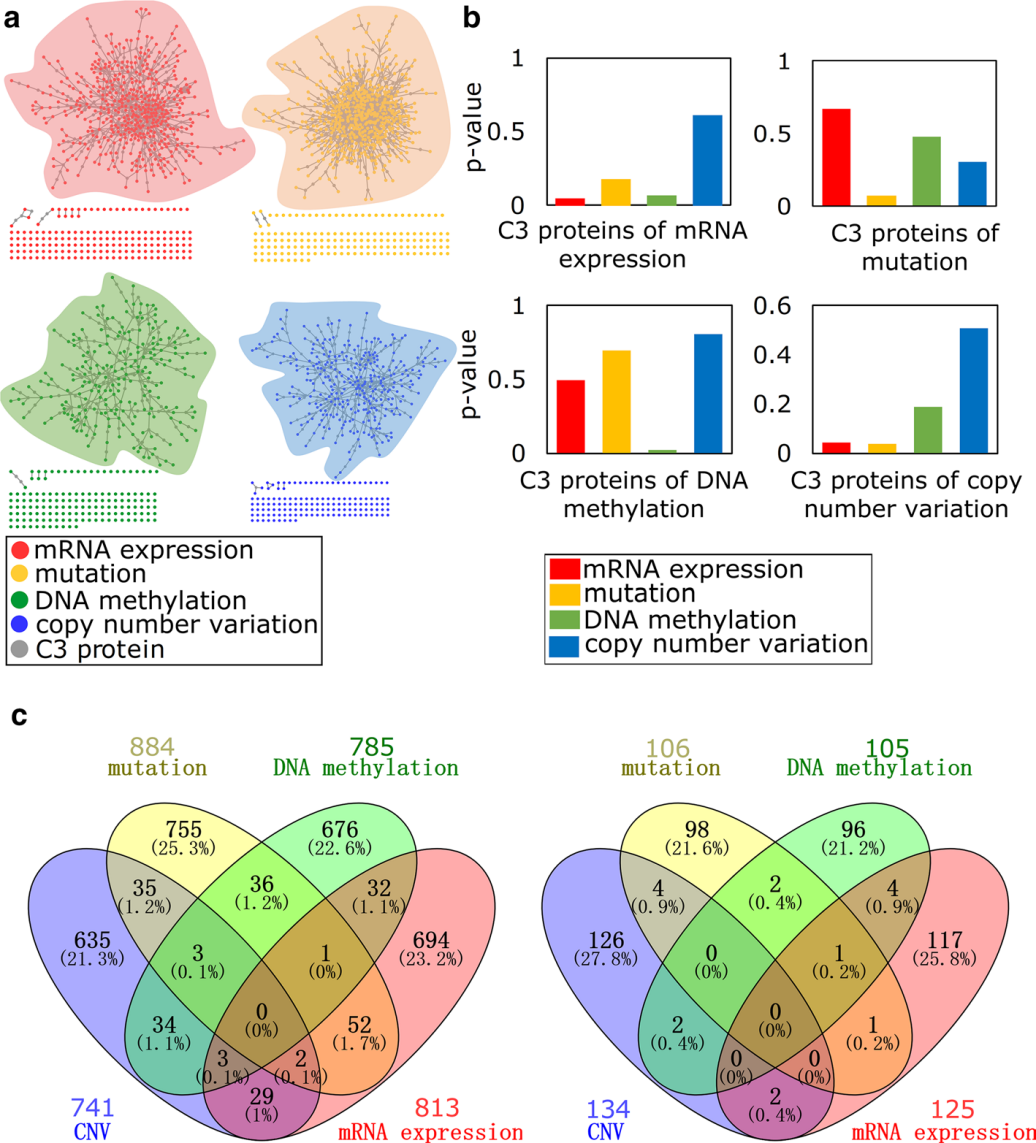
Specificity of C3 module in TCGA multi-omics data of BRCA. **a** The C3 module of four omics data of BRCA: (i) mRNA expression; (ii) mutation; (iii) DNA methylation; (iv) copy number variation. **b** Specificity of C3 proteins imported by different omics data. Red, yellow, green, and blue bars indicate the degree of perturbation of the C3 proteins imported by the four omics data, compared to the rest of the network (*p* value obtained by Wilcoxon test). **c** Venn diagram of different omics data (left) and four different C3 proteins we obtained (right)

C3 method can be used not only for DAPs, but also for the analysis of genome-wide to obtain genome-wide [16] disease neighborhoods. We collected four omics data: (1) mRNA expression; (2) mutation; (3) DNA methylation from TCGA and (4) copy number variation (see Methods), and successfully identified multi-omics neighborhoods of BRCA. In order to discover the connections between multi-omics data, we used the same human interactome network. For multi-omics data of BRCA, we identified the mutated genes and imported the encoded proteins as seeds into C3 method to obtain their respective C3 modules (Fig. 4a). Then, at different omics levels, we compared mRNA expression, mutation, DNA methylation of C3 proteins. We found that the C3 proteins imported from different omics data have definite omics specificity. For example, in mRNA expression omics, the mRNA expression of the imported C3 proteins is highly disturbed while its mutation and DNA methylation are not disturbed to a high degree. But the only thing that is special is for the C3 proteins imported by copy number variation, their mRNA expression and mutation levels are also high (Fig. 4b).

For the same disease, is there any potential association between four omics data? We further want to see the relationship of their respective C3 proteins, we used the overlap to represent. As a result, we found that there is no significant association between four omics data, although all are related to the same disease. The same for their respective C3 proteins, there is no overlap between them, which indicates that C3 proteins of the four omics data are also specific. That is, for BRCA, four specific omics data correspond to four specific groups of C3 proteins (Fig. 4c).

In Table 3, we validated biological significance of four groups of omics of C3 proteins of BRCA, we considered three verification datasets: (1) OMIM; (2) GWAS; (3) Drug-target, for each validation dataset, we used the Wilcoxon test for statistical significance analysis of enrichment. OMIM and GWAS databases can indicate the relationship between C3 proteins and diseases, and Drug-target database can show the relationship between C3 proteins and the drug target. We find that the C3 proteins corresponding to these four omics data have different degrees of enrichment significance in these three verification datasets, which indicates that the C3 proteins identified by our C3 method have rich biological associations, they may be drug target, they also may be pathogenic gene encodes annotated in OMIM and GWAS.

**Table 3:** Biological significance of four omics of C3 proteins of BRCA

Data (#proteins)	mRNA expression (125) #proteins (*p* value)	Mutation(106) #proteins (*p* value)	DNA methylation (105) #proteins (*p* value)	CNV(134) #proteins (*p* value)
OMIM(7952)	100(*0*)	75(*0.0056*)	85(*0*)	91(*0.01652*)
GWAS(5022)	61(*0.00371*)	57(*0.00022*)	50(*0.01305*)	53(0.2789)
Drug-target(2635)	40(*0.00035*)	32(*0.00328*)	38(*0.00002*)	34(*0.04129*)

PPI network: 13,460 proteins. The italics number means less than 0.05

**Fig. 5:**
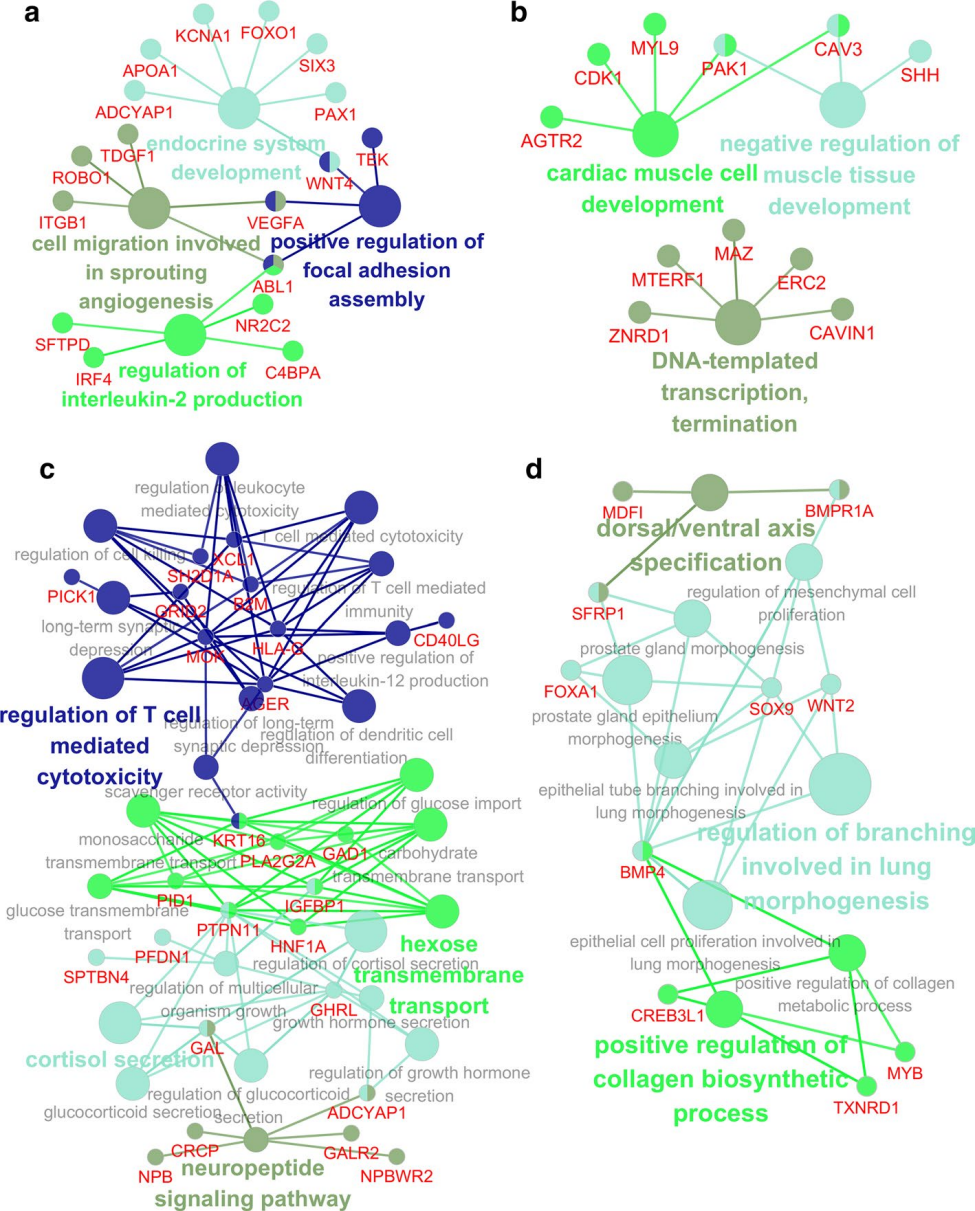
Biological significance of C3 proteins from four omics data of BRCA. GO term (BP) enrichment analysis for C3 proteins obtained from **a** mRNA expression, **b** mutation, **c** DNA methylation, and **d** copy number variation. We used tool ClueGO to get the functional-gene network of the results. GO terms were statistically significant enriched for a set of genes, names of which are highlighted in red, other different colors represent different GO term clustering and the color of gene nodes represents the associated GO term. Only the most prominent term name in each group is colored and the larger term nodes are, the higher the enrichment degree is

In order to further explicitly evaluate the biological functions of the C3 proteins, we used ClueGO [34] to conduct GO term (BP) enrichment analysis of these C3 proteins (*p* value < 0.01) of these C3 proteins (Fig. 5). And enriched GO terms are indispensable for the human, such as: development of endocrine system, development of cardiomyocytes, transport of glucose across membranes, and positive regulation of collagen metabolism. Many of them are also associated with BRCA, which again indicates that it is feasible to apply C3 in multi-omics data and our method can be used to identify different biologically active C3 proteins from different omics.

## Discussion

Disease module helps to understand the molecular mechanisms underlying diseases and identify novel drug targets. Topological properties of disease module in the human interactome are the basis for all network-based prioritization tools for studying human diseases. However, DAPs appear to form plenty of SCCs in the incomplete interactome. The problem we strive to solve is about how to determine their connectivity pattern and detect a concise disease module. To solve it, we proposed a C3 method to connect SCCs in the interactome by importing C3 proteins and C3 interactions with high connectivity. Our results highlight the role of C3 proteins and C3 interactions in connecting DAPs, which are dispersed as SCCs in incomplete interactome. Then we compared our method with DIAMOnD and random simulations to verify the succinctness and connectivity significance of the C3 module we obtained. We also got some unexpected results: the overlap between different omics data of the same disease is not high, which shows a clear omics specificity. From the perspective of biology, being the basis of disease characteristics, C3 proteins and C3 interactions may offer better and more accurate candidates. In the end, we hope that our method can help to study potential disease mechanisms, disease heterogeneity, drug response, capture novel pathways and genes.

## Conclusions

In this work, we develop C3 method, which is proved to be able to effectively construct succinct disease modules with relatively few C3 proteins for 299 diseases from OMIM and GWAS databases and multi-omics data of BRCA from TCGA. And the disease module helps us to understand the disease pathways and identify pathogenic genes.

## Methods

### Networks information

For the sake of accuracy of our experiment, we used the human interactome, which is also used in DIAMOnD [9]: It derives from seven sources of protein interactions by Ghiassian SD et al.: (1) regulatory interactions derived from transcription factors binding to regulatory elements; (2) binary interactions from several yeast-two-hybrid high-throughput data sets and literature-curated data sets; (3) literature-curated interactions derived mostly from low-throughput experiments; (4) metabolic enzyme-coupled interactions; (5) protein complexes; (6) kinase-substrate pairs; and (7) signaling interactions. The union of all interactions from (1) to (7) yielded a network of 13,460 proteins that were interconnected by 141,296 interactions [10]. In addition, we used three other networks to supplement the experiment: We took a current interactome from BioGRID (https://thebiogrid.org/) and used the latest release compiled on June, 2019 and only selected human interactions, finally we got 17,741 genes and 338,793 interactions. And in order to avoid getting the observed performance which is solely due to study biases in human interactome, we got networks from Bioplex (https://bioplex.hms.harvard.edu/) and HuRI (https://interactome.dfci.harvard.edu/H_sapiens/), finally Bioplex had 10,961 genes and 56,553 interactions and HuRI had 7352 genes and 38,414 interactions. Network data was included into Additional file 1.

### DAPs of 299 diseases

Menche et al. [10] integrated DAPs from OMIM (Online Mendelian Inheritance in Man; https://www.ncbi.nlm.nih.gov/omim) [22] which provides a link between genes and disease, and GWAS (Genome-Wide Association Study). The DAPs from GWAS were obtained from the Phenotype-Genotype Integrator database (PheGenI; www.ncbi.nlm.nih.gov/gap/PheGenI) [8] which merges GWAS catalog data with several databases housed at the NCBI (National Center for Biotechnology Information), and it supports searching annotated tables of genes based on phenotype. They used a genome-wide significance cutoff of *p* value ≤ 5.0e−8. Then in order to combine OMIM and GWAS they used the MeSH (Medical Subject Headings; https://www.nlm.nih.gov/mesh/) vocabulary. Using the hierarchical structure of the MeSH classification, in the result they found at least 20 DAPs and DAPs for which they have interaction information, and obtained 299 diseases and 3173 associated proteins.

### TCGA multi-omics data

We obtained publicly available The Cancer Genome Atlas (TCGA) multi-omics data of BRCA from UCSC Cancer Browser [35] (https://xena.ucsc.edu). For mRNA expression, we used an R packet edge [36] and took the threshold of |log(FC)| > 2 and adj. *p* value < 0.001 to get 860 mRNA expression genes, where 632 mapped to human interactome were taken as DAPs. We used TCGA BRCA somatic mutation data and chose 889 genes that mutation counts > 10 and mutation frequency > 0.01, where 689 mapped were taken as DAPs. DNA methylation values, described as beta values, we used an R packet limma [37] and choose 837 genes of the threshold |log(FC)| > 0.19 and adj. *p* value < 1.0e−14, and only 367 mapped were taken as DAPs. BRCA thresholded gene-level copy number variation (CNV) estimated values to -2, − 1, 0, 1, 2, representing homozygous deletion, single copy deletion, diploid normal copy, low-level copy number amplification, or high-level copy number amplification. Then we obtained 863 genes of threshold values − 2 and 2 and only 446 mapped were taken as DAPs. After processing the data, we obtained four altered data sets of multi-omics DAPs for BRCA, which are mRNA expression, mutation, DNA methylation and copy number variation.

### Biological validation data sets

Following data is provided in Additional file 1.

(i) Gene expression of asthma specific compiled from eight sources: we selected eight expression data of direct asthma relevance from the Gene Expression Omnibus (GEO; https://www.ncbi.nlm.nih.gov/geo). We analyzed samples from GSE470, GSE473, GSE3004, GSE16032, GSE18965, GSE31773, GSE4302, GSE2125 with GEO2R, a total of 4111 differentially expressed genes were obtained where only 3273 in human interactome.
(ii) Gene ontology term similarity (BP): To elucidate the biological processes associated with the DAPs, we used the GO biological process category. We calculated the GO term similarity (BP) of all genes, took the top 4000 proteins with high similarity to all DAPs, and only 3154 in human interactome. The calculation tool was R package GOSemSim [26]
(iii) Disgenet: Disgenet data of asthma got from DisGeNET database (https://www.disgenet.org/). Sources of data include literature data, inferred data, and animal models data, totally we got 1817 genes associate with the asthma.
(iv) OMIM: OMIM genes got from Online Mendelian Inheritance in Man (OMIM; https://omim.org), totally we got 9915 genes.
(v) GWAS: GWAS genes got from Genome-Wide Association Studies (GWAS; https://www.ebi.ac.uk/gwas), totally we got 15,858 genes.
(vi) Drug-target: Drug-target genes obtained from The Drug Gene Interaction Database (DGIdb; https://www.dgidb.org), totally we got 2993 genes

### C3 algorithm

A basic detection process of C3 method is as follows: For a specific disease, we mapped its DAPs (seeds set *D*) into the network $G(N,E)$, where *N* is the proteins set and *E* is the interaction set. *ND* is the set of non-seeds, ${n}_{0}=\left|ND\right|$ and |$\bullet$| is the size of the corresponding set [38, 39].

**Export SCCs (ExScc):**
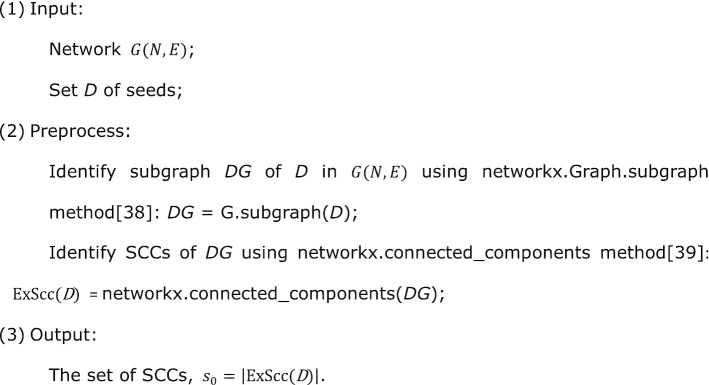


Then we considered the immediate neighbors of *D* as the candidate proteins, and the adjacent edges of *D* as the candidate interactions.

**Connectivity significance:**

For a candidate protein *i*, its connectivity degree to SCCs ${k}_{s}$ is defined as:

1 $${k}_{s}=\left|\left(s|s\in S,\exists\, \hbox{a\, node} \,l\, \hbox{of} \,s,(i,l)\in E\right)\right|$$

and its connectivity degree to non-seeds ${k}_{nd}$ is defined as:

2 $${k}_{nd}=\left|\left(j|j\ne i,j\in ND,\left(i,j\right)\in E\right)\right|$$

Overall, the connectivity degree of *i* is calculated as $k={k}_{s}+{k}_{nd}$.

Analogously, for a candidate interaction $e(p,q)$ between protein *p* and *q*, we quantified the ${k}_{s}$ and ${k}_{nd}$ for *e* as:

3 $${k}_{s}=\left|\left(s|s\in S,\exists\, \hbox{a\, node \,}l \,\hbox{of} \,s,\left(p,l\right)\in E\, \hbox{or }\,\left(q,l\right)\in E\right)\right|$$

4 $${k}_{nd}=\left|\left(j|j\ne p,j\ne q,j\in ND,\left(p,j\right)\in E\, \hbox{or }\,\left(q,j\right)\in E\right)\right|$$

The connectivity degree of *e* is also calculated as $k={k}_{s}+{k}_{nd}$.

Then, for randomly scattered SCCs, the connection probability that a candidate protein or a candidate interaction with a total connectivity degree *k* has exactly *k*_*s*_ links to SCCs is given by the hypergeometric [40]:

5 $$p\left(k,{k}_{s}\right)=\frac{\left(\begin{array}{c}{s}_{0}\\ {k}_{s}\end{array}\right)\left(\begin{array}{c}{n}_{0}\\ {k}_{nd}\end{array}\right)}{\left(\begin{array}{c}{s}_{0}+{n}_{0}\\ k\end{array}\right)}$$

To evaluate whether a candidate protein or a candidate interaction has more connections to SCCs than expected under null hypothesis, we calculate the connectivity significance *p* value, i.e. the cumulative probability for the observed or any higher connectivity degree to SCCs:

6 $$p\, \hbox{value}\,\left(k,{k}_{s}\right)= \sum_{{k}_{i}={k}_{s}}^{k}p(k,{k}_{i})$$

This *p* value is used as a connectivity significance measure, and is used to quantify the abilities of a candidate protein and a candidate interaction in connecting SCCs.

**C3 protein and C3 interaction:**

Then, we got the C3 disease module through a greedy process. In each iteration, *p* value < 0.05 and the lowest protein is selected as the C3 protein to connect SCCs and be added to *D*, then update $\text{ExScc}({\mathrm{D}})$. When ${s}_{0}$ is unchanged, *p* value < 0.05 and the lowest interaction is selected as the C3 interaction to connect SCCs and two proteins that occupy the endpoints of it, both are added to *D*, then update $\text{ExScc}({\mathrm{D}})$. Repeated these steps, when ${s}_{0}$ is unchanged, i.e. there are no C3 protein and C3 interaction that can connect SCCs with *p* value < 0.05, the process stopped, we obtained the C3 disease module.

The C3 proteins and the pair of proteins occupying the endpoints of C3 interactions are defined as intermediate proteins, which are collectively referred to as “*C3 proteins*” in the functional analysis.

**Pseudocode:**
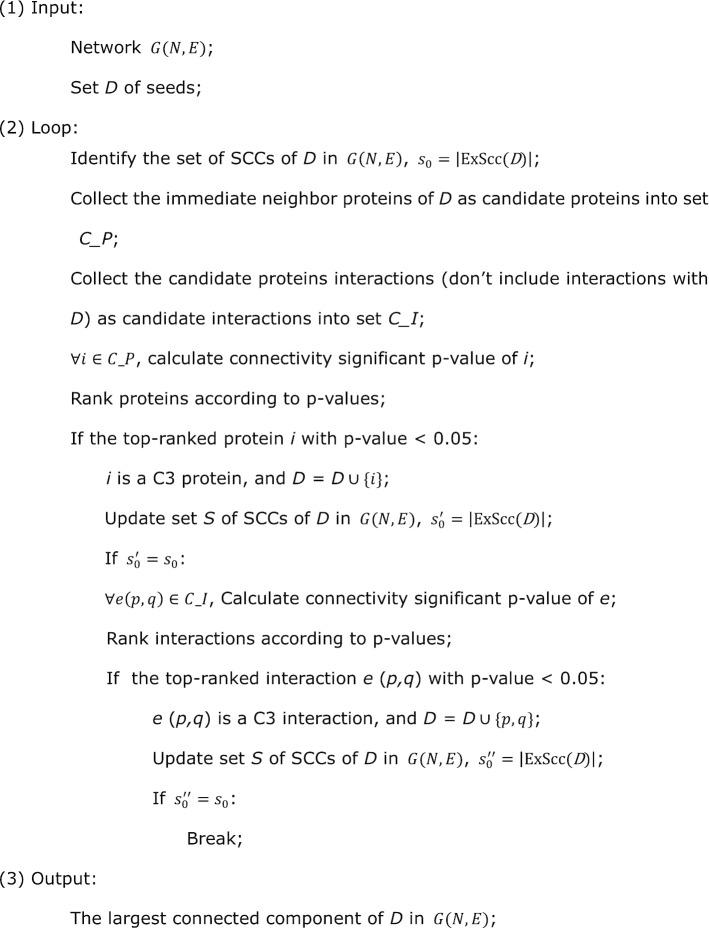


The largest connected component of *D* in $G(N,E)$ is the disease module. Its time complexity is $O({n}^{2})$. We implemented C3 with Python and the codes can be downloaded from https://github.com/wangbingbo2019/C3.

### Randomized experiments

For asthma, we conducted two randomized experiments. One is completely random, namely when we randomly selected 100 proteins from the network (the number consistent with DIAMOnD) using numpy.random.choice method [41] and added them to seed set *D*, we treated the largest connected component of *D* as the random module. After 1000 random times, we got the mean $\upmu$ and standard deviation $\upsigma$ of the number of connected seeds in random modules. Z-score of true (C3 or DIAMOnD) disease module with *T* (#DPcC or #DPcD) connected seeds is given by the following:

7 $$z\,\hbox{score}= \frac{T-\mu }{\sigma }$$

The other is that instead of randomly selecting from the network, we randomly selected 100 proteins from the immediate neighbors of *D* and added them to seed set *D* to get the final random module. After 1000 random times, z-score is also calculated by Eq. (7).

For BRCA, we randomly selected 40 random proteins matching size and degree distribution as BRCA seeds using numpy.random.choice method [41] for random experiments. After 1000 random times, we got the mean and standard deviation of the number of cP and iP in random modules. Z-score of C3 disease module with true number of cP and iP is also given by Eq. (7).

## Supplementary information

**Additional file 1:** Supplementary data and results.

## Data Availability

All supporting files can be downloaded from https://github.com/wangbingbo2019/C3 including: the networks data, disease genes from OMIM and GWAS, some data sets for validation, the C3 tool in Python, and the results of C3 modules.

## Abbreviations

C3: Connect separate connected components
SCCs: Separate connected components
DAPs: Disease-associated proteins
OMIM: Online Mendelian Inheritance in Man
GWAS: Genome-Wide Association Study
LCC: Largest connected component
TCGA: The Cancer Genome Atlas
BRCA: BReast CAncer
DPcD: Disease proteins contained in DIAMOnD module
DPcC: Disease proteins contained in C3 module
cP: Connected proteins
iP: Intermediate proteins
